# Epidemiological Characteristics and Survival Studies of Rhabdomyosarcoma in East Egypt: A Five-Year Multicenter Study

**DOI:** 10.5402/2012/674523

**Published:** 2012-05-17

**Authors:** M. A. Badr, Y. A. Al-Tonbary, A. K. Mansour, T. H. Hassan, M. R. Beshir, A. Darwish, R. A. El-Ashry

**Affiliations:** ^1^Pediatric Hematology/Oncology Units, Zagazig University, Zagazig 44111, Egypt; ^2^Pediatric Hematology/Oncology Units, Mansoura University, Mansoura 35516, Egypt

## Abstract

*Background*. Rhabdomyosarcoma (RMS) is the most common soft tissue sarcoma in children, it represents 5–8% of childhood malignancies. *Aim of the Work*. To evaluate the epidemiological characteristics and treatment outcome in two pediatric oncology centers. *Patients and Method*. A retrospective analysis was performed on 41 medical records of children with RMS during 6 years period. *Results*. The median age of patients was 6 years with 80.4% below 10 years. Head and neck was the most common primary site. Embryonal RMS was the most frequent histopathologic subtype. Stage IV was the most frequent stage. According to IRS postsurgical grouping classification, group 4 was the most frequent group. There was a significant relationship between histopathologic subtypes of tumor and metastasis, primary site of tumor and histopathologic subtype, age, metastasis, IRS presurgical stage and IRS postsurgical group and outcome. The overall survival rate was 56.9% ± 8.4 and the failure free survival rate was 68.3% ± 7.6. *Conclusion*. The epidemiological characteristics of our patients are quite near to the worldwide data, apart from the higher prevalence of stage IV and group 4 with most of the primary tumor site in the extremities. CWS2002 protocol of therapy had led to improvement in the curability of the disease.

## 1. Introduction

Rhabdomyosarcoma (RMS) accounts for approximately 3.5% of the cases of cancer among children aged 0 to 14 years and 2% of the cases among adolescents and young adults aged 15 to 19 years [1]; The incidence is 4.5 per million children and 50% of cases are seen in the first decade of life [2]. Approximately, 65% of cases are diagnosed in children less than six years of age with remaining cases noted in the 10-to-18-year-old age group. There is a slight predilection for disease in males, with a male to female ratio of 1.3 : 1 [3]. Several distinct histologic groups have prognostic significance, including embryonal rhabdomyosarcoma (ERMS), which occurs in 55% of patients; the botryoid variant (5%); alveolar rhabdomyosarcoma (20%); undifferentiated sarcoma (UDS) in 20% of patients [4]. Distinctive features appear to cluster around the site of the primary tumor, the age at diagnosis, and the histologic subtype. Head and neck RMS are more common in younger children, with orbital tumors being characterized by embryonal histology in most of cases. Extremity tumors are more common in adolescents and are more likely to have an alveolar histologic subtype. Nearly 80% of genitourinary tract RMS are embryonal in nature [5]. The most common sites are the head and neck (40%), followed by genitourinary tract (29%), extremities (14%), trunk (12%), and other sites in less than 5% of patients [6]. In patients with localized disease, overall 5-year survival rates have improved to more than 80% with the combined use of surgery, radiotherapy, and chemotherapy [7]. However, in patients with metastatic disease, little progress has been made in survival rates, with a 5-year event-free survival rate less than 30%. Those patients with metastatic disease without other high-risk factors including unfavorable site, more than 3 sites, bone marrow involvement, and age younger than 1 year or older than 10 years, have a better prognosis [8]. 

## 2. Patients and Methods

A retrospective analysis was performed on 41 medical records of children with RMS who were admitted, treated, and followed up at the hematology/oncology units of pediatric departments, Zagazig and Mansoura University children hospitals during the period from June 2004 to June 2009. Patients were followed up to September 2010. The followup period ranged from 5 to 74 months with a mean of 20 months. The medical records were reviewed for

- personal data for example, name, age, sex, consanguinity, and residence,
- presenting symptoms and signs,
- primary site of the tumor,
- histopathological characters of the tumor,
- routine laboratory investigations at presentation and during treatment, for example, complete blood count, liver function tests, kidney function tests, serum electrolytes, LDH, and alkaline phosphatase,
- imaging studies, for example, plain X-ray, ultrasound, CT, and MRI, on primary site and other common metastatic sites,
- risk stratification for patients treated according to VAC protocol and those treated according to the German pediatrics soft tissue study group (CWS) protocol [9],
- treatment protocols including surgery, radiotherapy, and chemotherapy,
- patients' outcome.

## 3. Results

Our study showed that the median age of our patients was 6 years with 80.4% of patients were below the age of 10 years. The male to female ratio was 1.15 : 1. Head and neck was the most common affected primary site of tumor followed by extremities, then genitourinary, and lastly the retroperitoneum (Table 1).

The embryonal RMS was the most frequent histopathologic subtype, followed by alveolar (28.6%), and lastly the botryoid and spindle subtypes in 4.7% for each (Table 2).

Tumor size more than 5 cm was present in 65.9% of patients, 12% of patients had lymph node involvement and 44% of patients had metastasis at time of diagnosis (Table 3).

Stage IV was the most frequent stage of our patients (43.9%), followed by stage III (29.3%), then stage I (17.1%), and lastly stage II (9.7%). According to IRS postsurgical grouping classification, group 4 was the most frequent group (43.9%), followed by group 1 (26.8%), then group 3 (19.5%) and lastly group 2 (9.8%).

There was a significant relationship between histopathologic subtypes of tumor and metastasis, all patients with alveolar subtype had metastasis at time of diagnosis while only 31% of embryonal subtype had metastasis.

There was highly significant (*P* < 0.001) statistical relationship between primary site of tumor and histopathologic subtype. All patients with head and neck RMS and 50% of both genitourinary and retroperitoneum RMS were of embryonal subtype while 58.3% of extremities RMS were of alveolar subtype (Table 4).

There was no significant relationship between primary site of tumor and metastasis (Table 4).

There was no significant statistical relationship between primary site of tumor and age (*P* = 0.4).

The relations between outcome and each of age, sex, primary site, histopathology, metastasis, IRS postsurgical grouping, IRS presurgical staging, protocol of treatment, and radiotherapy are shown in Tables 5 and 6.

There was a significant relationship between age and outcome of patients (*P* = 0.02). Seventy five percent of patients more than 10 years died while about 70% of patients less than 10 years survived (Table 5).

There was a significant relationship between metastasis and outcome (*P* = 0.0013), where 66.7% of patients who had metastasis at time of diagnosis died while 82.6% of patients without metastasis survived (Table 5).

There was a significant relationship between IRS postsurgical group and outcome, as 81.8% of group 1 and 100% of group 2 survived while 61.1% of group 4 died (Table 6).

There was a significant relationship between IRS presurgical stage and outcome, as 100% of stage I survive while 80% of stage IV died (Table 6).

There was a significant relationship between outcome and protocol of treatment and radiotherapy. Patients who treated with CWS protocol had a significantly better prognosis (*P* = 0.03) while patients who received radiotherapy had a significantly worse prognosis (*P* = 0.02) (Table 6).

**Figure 1:**
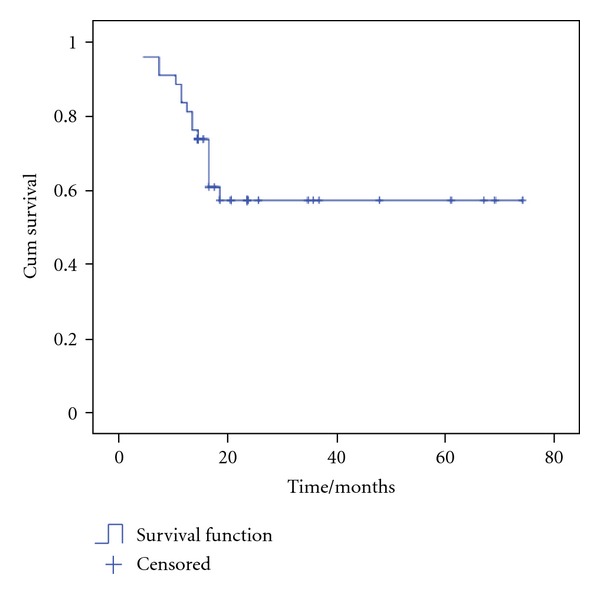
Overall survival (OS) of patients. The estimated OS rate was 56.9%  ±  8.4 with mean OS time of 47.8 months ±5.

**Figure 2:**
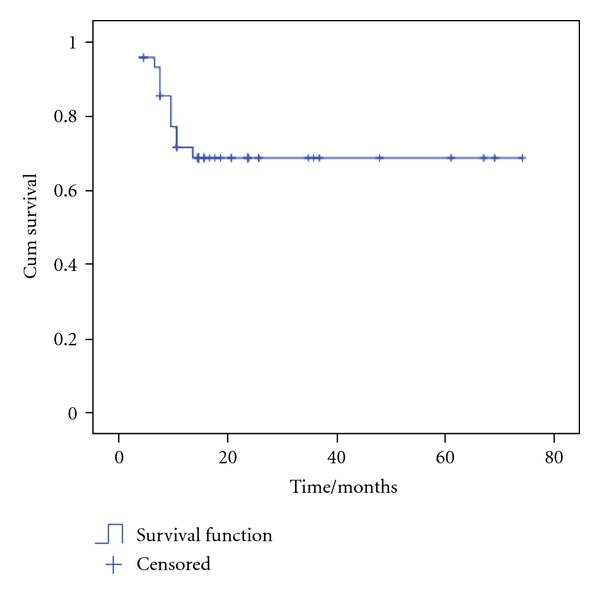
Failure-free survival (FFS) of patients. The estimated FFS rate was 68.3%  ±  7.6 with mean FFS time of 53.3 months ±4.9.

The estimated overall survival (OS) rate was 56.9%  ±  8.4 with mean OS time of 47.8 months ±5 (Figure 1) and the estimated failure free survival (FFS) rate was 68.3%  ±  7.6 with mean FFS time of 53.3 months ±4.9 (Figure 2).

**Figure 3:**
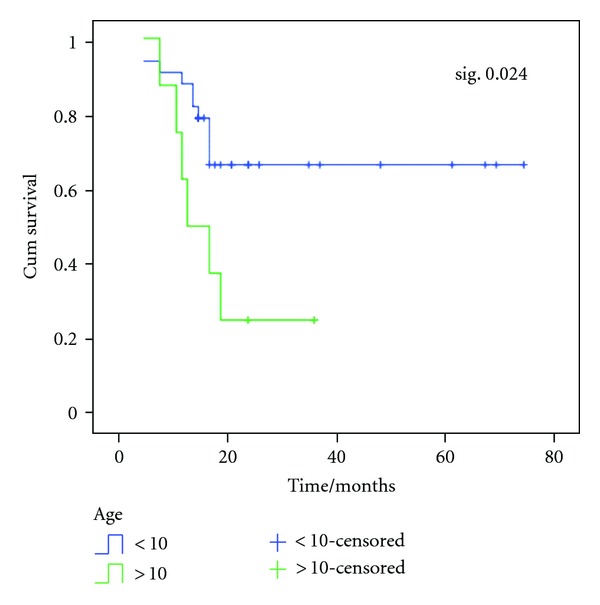
OS according to age of patients. The estimated OS rate was 66.3%  ±  8.9 and 25%  ±  15.3 for patients < 10 years and ≥ 10 years old respectively, while mean OS time was 53.4 months ±5.4 and 19 months ±3.6 for patients < 10 years and ≥ 10 years old respectively.

The estimated OS rate was 66.3%  ±  8.9 and 25%  ±  15.3 for patients < 10 years and ≥ 10 years old, respectively, while mean OS time was 53.4 months ±5.4 and 19 months ±3.6 for patients < 10 years and ≥ 10 years old, respectively (Figure 3).

**Figure 4:**
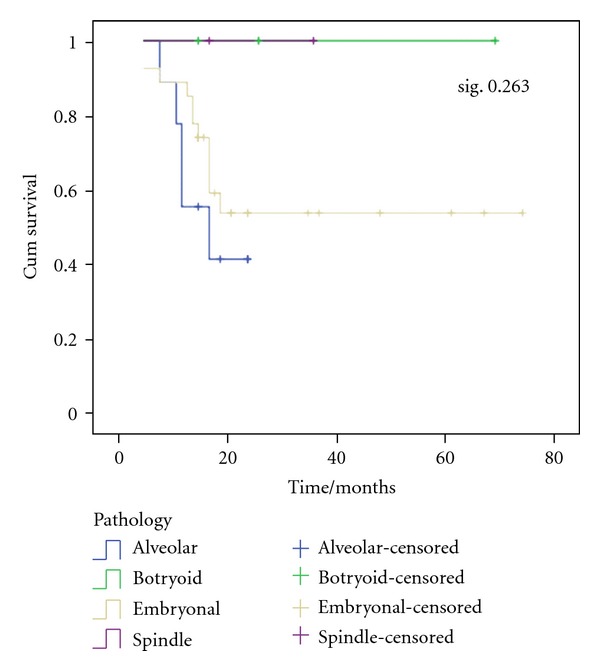
OS according to histopathologic subtypes of tumor. The estimated OS rate was 53.9%  ±  10.6 and 41.7%  ±  17.3 for patients with embryonal and alveolar rhabdomyosarcoma, respectively.

The estimated OS rate was 53.9%  ±  10.6 and 41.7%  ±  17.3 for patients with embryonal and alveolar rhabdomyosarcoma, respectively (Figure 4).

**Figure 5:**
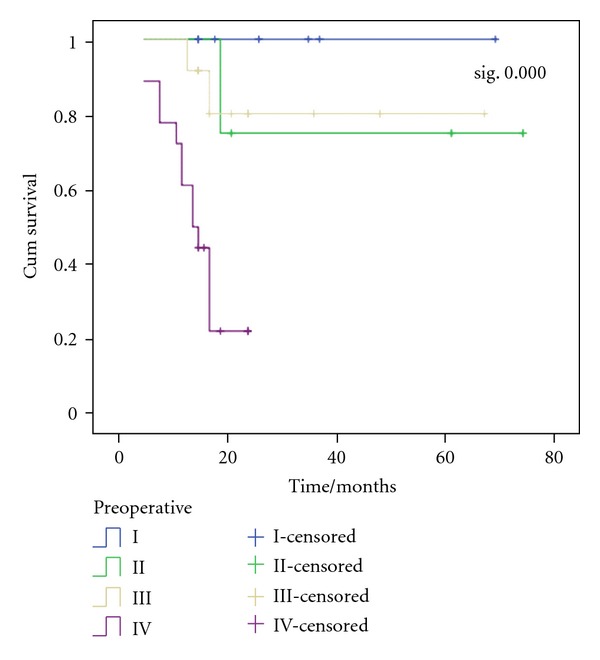
OS according to IRS presurgical stages of patients. The estimated OS rate was 75%  ±  21, 80.2%  ±  12.8, and 22.2%  ±  10.8 for patients with stage II, stage III, and stage IV, respectively.

The estimated OS rate was 75%  ±  21, 80.2%  ±  12.8, and 22.2%  ±  10.8 for patients with stage II, stage III, and stage IV, respectively (Figure 5).

**Figure 6:**
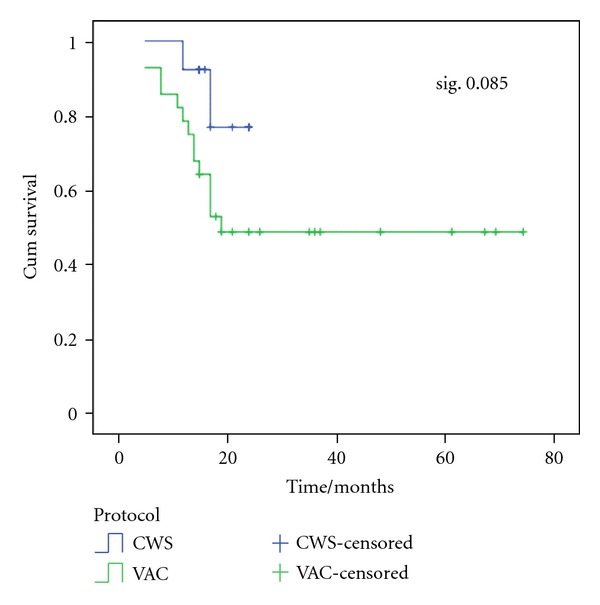
OS according to protocols of treatment. The estimated OS rate was 48.9%  ±  9.6 and 76.9%  ±  15.3 for patients on VAC and CWS protocol, respectively. While mean OS time was 42.6 months ±5.9 and 22 months ±1.7 for patients on VAC and CWS protocol, respectively.

The estimated OS rate was 48.9%  ±  9.6 and 76.9%  ±  15.3 for patients on VAC and CWS protocol, respectively. While mean OS time was 42.6 months ±5.9 and 22 months ±1.7 for patients on VAC and CWS protocol, respectively (Figure 6).

## 4. Discussion

Rhabdomyosarcoma (RMS) is a highly malignant childhood cancer. It is the most common form of soft tissue sarcoma in the first two decades of life, with a peak incidence in very young children [1]. The median age of our patients was 6 years with 80.4% of patients were below the age of 10 years. These results are similar to another Egyptian study conducted by Shouman et al. [10] who reported the same median age but with 60% of patients below the age of 10 years. The IRS IV reported that the median age of patients was 5-year, with 72% of patients below the age of 10 years [11].

In our study, 53.6% of patients were males while 46.4% were females with male to female ratio of 1.15 : 1. IRS IV reported higher male to female ratio (1.6 : 1) [11].

In our study, head and neck was the most common affected primary site of tumor (36.6%), followed by extremities (29%), then genitourinary (19.6%), and lastly retroperitoneum in (14.6%). These results are different from Abd El-Aal et al. who reported that the genitourinary is the second most common affected site (23.6%), after head and neck (36.4), followed by extremities (16.3), then retroperitoneum (12.7) [12]. Also the IRS IV found that head and neck was the most common affected primary site of tumor (41%), followed by the genitourinary site (31%), then extremities (13%), and retroperitoneum (7%) [11]. This difference can be explained by small number of our patients compared to these studies.

In our study, embryonal RMS was the most frequent histopathologic subtype (61.9%) while alveolar RMS represents 28.6% of patients. Hessissen et al. [13] found that embryonal subtype represents 73% while alveolar subtype represents 13% of patients and Abd El-Aal et al. [12] found that embryonal and alveolar subtypes represent 87.3% and 12.7% of patients, respectively. The IRS IV reported that the embryonal subtype represent 70% including the botryoid and spindle cell variants, this is quite near to our results if the botryoid and spindle cell variants were added to embryonal subtype (71.7%) [11]. 

In our study, 65.9% of patients had tumor size more than 5 cm at time of diagnosis while 34.1% had tumor size below 5 cm. These results are lower than the 75% reported by a Japanese study conducted by Hosoi et al. [14], and higher than the 51% and 55% reported by the IRS IV and Abd El-Aalet al., respectively, for patients who had tumor size more than 5 cm [11, 12].

In our study, 12.2% of patients had lymph node involvement at time of diagnosis. This result is similar to Shouman et al. [10] and the IRS IV [11] who found that 15% of patients had LN involvement. Hosoi et al. [14] showed that 19% of patients had LN involvement. Our study showed that 43.9% of patients had metastasis at time of diagnosis. Koscielniak et al. [15] reported that fewer than 25% of patients have metastatic disease at diagnosis. Also, 63% of patients received radiotherapy, this result is the same result reported by Shouman et al. [10].

In our study, according to IRS postsurgical grouping classification, group 4 was the most frequent group (43.9%), followed by group 1 (26.8%), then group 3 (19.5%), and lastly group 2 (9.8%). These results are different from Hessissen et al. [13] who reported that group 3 was the most frequent group (51%) then group 2, 1, and 4 (22%, 14%, and 13% of patients, resp.). Shouman et al. [10] and Hosoi et al. [14] reported the same order of frequency as Hessissen et al.

Regarding the IRS presurgical staging classification, stage IV was the most frequent stage (43.9%) of our patients, followed by stage III (29.3%), then stage I (17.1%), and lastly stage II (9.7%). These results differ from Shouman et al. [10] who reported that stage III was the most frequent stage (46%), followed by stage IV (24%), then stage I (19%), and lastly stage II (11%). Hosoi et al. [14] reported the same order of frequency as Shouman et al. while Abd El-Aal et al. [12] founded that stage II was the most frequent stage, followed by stage III, then stage IV, and lastly stage I.

High percentage of patients with metastasis at time of diagnosis, group 4 and stage IV in our patients can be explained by the unawareness of primary health care physicians about early presenting symptoms and signs of the disease, together with the unavailability of diagnostic facilities which can allow earlier picking up of cases with localized disease.

In our study, there was a significant statistical relationship between histopathologic subtypes of tumor and metastasis, all patients with alveolar subtype had metastasis at time of diagnosis while only 31% of embryonal subtype had metastasis.

In our study, there was no significant relationship between primary site of tumor and age (*P* = 0.4). Wiener [5] stated that head and neck RMS are more common in younger children; on the other hand, extremities RMS are more commonly found in adolescents.

Our results showed that there was highly significant (*P* < 0.001) relationship between primary site of tumor and histopathologic subtype. All patients with head and neck RMS and 50% of both genitourinary and retroperitoneum RMS were of embryonal subtype, while 58.3% of extremities RMS were of alveolar subtype. Wiener [5] found that head and neck RMS are being characterized by embryonal histology in most cases; on the other hand, extremities RMS are more likely to have an alveolar subtype and nearly 80% of genitourinary tract RMS are embryonal in nature. Lawrence et al. [16] reported that head and neck RMS are most commonly of the embryonal subtype. Mandell et al. [17] reported that nearly 50% of extremities RMS are of the alveolar subtype.

In our study, there was a significant relationship (*P* = 0.02) between age and outcome of patients. Seventy five percent of patients more than 10 years died while about 70% of patients less than 10 years survived. Our results are in agreement with Punyko et al. [7] who found that patients aged 1–9 years at time of diagnosis showed good prognosis, while those below 1 year and 10–19 years showed poor prognosis. On the other hand, our results revealed no significant statistical relationship between primary site of tumor and outcome. These results are not matched with Crist et al. [11] who reported that primary sites with more favorable prognosis include the orbit and nonparameningeal head and neck, paratestis, vulva, vagina, uterus, and biliary tract.

In our study, there was no significant relationship between histopathologic subtype of tumor and outcome (*P* > 0.05). The IRS-IV did not include histology as an independent prognostic factor. There is evidence to suggest that site, which is associated with histopathologic subtype, is an independent prognostic factor, and that histology is a prognostic factor only because of its association with site [18].

Our study reported that there was a significant relationship (*P* = 0.0013) between metastasis and outcome, where 66.7% of patients who had metastasis at time of diagnosis died while 82.6% of patients without metastasis survived. Breneman et al. [19] found that children with metastatic disease at diagnosis have the poorest prognosis and the prognostic significance of metastatic disease is modified by tumor histology (embryonal is more favorable than alveolar) and by the number of metastatic sites.

 In our study, 5-year OS was 56.9%. This result is quite near to the 50% 5-year OS reported by Shouman et al. [10] but lower than the 74% reported by Abd El-Aal et al. [12]. Hessissen et al. [13] reported 10 years OS of 70%. Hosoi et al. [14] reported 69% 3-year OS and 61% 5-year OS.

In our study, 5-year FFS was 68.3%. This result is similar to that reported by Abd El-Aalet al. (68%) [12] but higher than the 40% 5-year FFS reported by Shouman et al. [10].

In our study, the estimated OS in relation to age was higher in patients < 10 years than in patients ≥10 years (66.3% versus 25%). Abd El-Aal et al. [12] reported that OS was 56% and 46% for patient < 10 and ≥ 10 years old, respectively.

Our results showed that the estimated OS in relation to histopathologic subtypes was higher in embryonal subtype than in alveolar subtype (53.9% versus 41.7%). Abd El-Aal et al. [12] reported that 5-year OS was 80% and 65% for embryonal and alveolar subtype, respectively, while Pappo et al. [4] reported that 5-year OS was 64% and 26% for embryonal and alveolar subtype, respectively.

In relation to the IRS presurgical staging classification, our results showed that the highest estimated OS was stage I (100%) followed by stage III (80.2%), then stage II (75%), and lastly stage IV with (22.2%). Hosoi et al. [14]. found that the estimated OS was highest in stage I followed by stage II then stage III and lastly stage IV with 79%, 77%, 59%, and 36% for stage I, II, III, and IV, respectively.

Since 2008, CWS 2002 protocol was selected for treatment of soft tissue sarcomas in our unit. Our study showed that there was a significant statistical relationship (*P* = 0.03) between protocol of treatment and outcome, where 84.6% of patients who received CWS survived versus 50% of those who received VAC protocol. The estimated OS was higher in patients who received CWS protocol than those who received VAC protocol (76.9 versus 48.9%). Also, the estimated FFS was higher for patients who received CWS protocol than those who received VAC protocol with 76.9% and 63.4% for CWS and VAC protocols, respectively.

## 5. Conclusion

Apart from the higher prevalence of stage IV and group 4 in our patients and the higher percentage of patients with primary tumor site in the extremities, the epidemiological characteristics of our patients are quite near to the worldwide data. The application of the intensive-risk-based CWS2002 protocol for treating our patients had led to improvement in the curability of the disease.

## Figures and Tables

**Table 1:** Demographic characteristics and the primary tumor sites of patients.

	*n* = 41	%
Age (years)		
$\overset{®}{\text{X}}\pm \text{SD}$	6.3 ± 4.3	
Range	(1–16)	
<10	33	80.4
≥10	8	19.6

Gender		
Male	22	53.6
Female	19	46.4

Primary site		
Head and neck	15	36.6
Extremities	12	29.2
Genitourinary	8	19.6
Retroperitoneum	6	14.6

**Table 2:** Histopathological subtypes of tumor.

Histopathology	*n* = 41	%
Embryonal	27	65.9
Alveolar	9	21.9
Botryoid	3	7.3
Spindle cell	2	4.9

**Table 3:** Tumor size, lymph node involvement and distant metastasis of patients.

	*n* = (41)	%
Tumor size (cm)		
<5	14	34.1
>5	27	65.9

Lymph node involvement		
−ve	36	87.8
+ve	5	12.2

Distant metastasis		
−ve	23	56.1
+ve	18	43.9

**Table 4:** Relationship between primary site of tumor, histopathology, and metastasis.

	Head and neck		Extremities		Genitourinary		Retroperitoneum		*x* ^²^	*P* value
	*n* = 15	%	*n* = 12	%	*n* = 8	%	*n* = 6	%		
Histopathology										
Embryonal (*n* = 27)	15	100	5	41.7	4	50.0	3	50.0	26.59	<0.001**
Alveolar (*n* = 9)	0	0	7	58.3	0	0	2	33.3	21.37	<0.001**
Others (*n* = 5)	0	0	0	0	4	50.0	1	16.7	14.32	0.002*

Metastasis										
−ve	10	62.5	5	28.6	4	75	4	50	2.09	0.55
+ve	5	37.5	7	71.4	2	25	4	50		

**: highly significant; *: significant.

**Table 5:** Relation between outcome and age, sex, primary site, histopathology, and metastasis.

		Survivors		Dead		*x* ^²^	*P *value
		*n* = 25	%	*n* = 16	%		
Age (years)							
< 10	*n* = 33	23	69.7	10	30.3	5.27	0.02*
≥10	*n* = 8	2	25	6	75		

Gender							
Male	*n* = 22	12	54.5	10	45.5	0.83	0.36
Female	*n* = 19	13	68.4	6	31.6		

Primary site							
Head and neck	*n* = 15	7	46.7	8	53.3	2.57	0.1
Extremities	*n* = 12	8	66.7	4	33.3	0.02	0.88
Genitourinary	*n* = 8	6	75.0	2	25	0.58	0.44
Retroperitoneum	*n* = 6	4	66.7	2	33.3	0.09	0.76

Histopathology							
Embryonal	*n* = 27	15	55.6	12	44.4	0.98	0.32
Alveolar	*n* = 9	5	55.6	4	44.4	0.14	0.72
Others	*n* = 5	5	100	0	0	2.02	0.15

Metastasis							
−ve	*n* = 23	19	82.6	4	17.4	10.3	0.0013*
+ve	*n* = 18	6	33.3	12	66.7		

*: significant.

**Table 6:** Relationship between outcome and IRS postsurgical groups, IRS presurgical stages, protocol of treatment, and radiotherapy.

		Survivors		Dead		*x* ^²^	*P *value
		*n* = 25	%	*n* = 16	%		
IRS postsurgical group							
1	*n* = 11	9	81.8	2	18.2	1.68	0.19
2	*n* = 4	4	100	0	0	1.31	0.25
3	*n* = 8	5	62.5	3	37.5	0.02	0.88
4	*n* = 18	7	38.9	11	61.1	7.32	0.0072*

IRS presurgical stage							
I	*n* = 7	7	100	0	0	5.27	0.02*
II	*n* = 4	2	50	2	50	0.22	0.63
III	*n* = 12	9	75	3	25	1.09	0.29
IV	*n* = 18	7	38.9	11	61.1	7.22	0.0012*

Protocol of treatment							
VAC	*n* = 28	14	50	14	50	4.47	0.03*
CWS	*n* = 13	11	84.6	2	15.4		

Radiotherapy							
Yes	*n* = 26	12	46.2	14	53.8	6.56	0.01*
No	*n* = 15	13	86.7	2	13.3		

*: significant.
